# Development of an Enzyme-Linked Immunosorbent Assay for Dibutyl Phthalate in Liquor

**DOI:** 10.3390/s130708331

**Published:** 2013-06-27

**Authors:** Hua Kuang, Liqiang Liu, Liguang Xu, Wei Ma, Lingling Guo, Libing Wang, Chuanlai Xu

**Affiliations:** 1 State Key Laboratory of Food Science & Technology, School of Food Science & Technology, Jiangnan University, Wuxi 214122, China; E-Mails: xuliguang2006@126.com (L.X.); mawei209@126.com (W.M.); raxray@gmail.com (L.L.); gling0329@126.com (L.G.); xcl@jiangnan.edu.cn (C.X.); 2 Research Centre of Hunan Entry–Exit Inspection and Quarantine Bureau, Changsha 410001, China; E-Mail: wanglb1@126.com

**Keywords:** di-*n*-butyl phthalate, ELISA, liquor, monoclonal antibody

## Abstract

A monoclonal antibody specifically recognizing dibutyl phthalate (DBP) was prepared based on a hapten (di-*n*-butyl-4-aminophthalate). After optimizing various parameters such as concentrations of antibody, coating antigen and composition of the assay buffer, an inhibition curve was plotted with the 50% inhibition concentration value (IC_50_) 33.6 ± 2.5 ng/mL. A low level of cross-reactivity (<5%) was found for other phthalate esters. Recovery tests were conducted using liquor simulant (a mixture of water and ethanol) at two fortification levels (100 ng/mL and 300 ng/mL). The recovery rates ranged from 84.7% to 94.5% with a coefficient of variation between 7.1% and 12.8%. Nine liquor samples of different alcoholic strengths were detected using the proposed measure and confirmatory analysis was performed using liquid chromatography-mass spectroscopy (LC-MS). The detection results showed good consistency between the two measures and all the data above indicated that the proposed ELISA could be applied in DBP screening.

## 1. Introduction

Phthalate esters are in widespread use as plasticizers in the production of various consumable or household products. Dibutyl phthalate (DBP), a major phthalate ester, is used to produce epoxy resins, cellulose esters and special adhesive formulations. Worldwide production of phthalate esters and their frequent application in a wide range of products in daily use has resulted in their presence in all parts of the environment and, consequently, in food. It has been reported that DBP accounts for up to 58.7% of phthalate ester pollution measured in the atmosphere of Nanjing metropolitan area of China [1]. Many studies have confirmed that DBP and other phthalate esters could migrate from food packaging materials into the food itself [2–4]. In fact, DBP was recently reportedly found in liquor [5,6], juice [7], vegetable oil [8], milk [9] and other foods [10]. The phthalate ester family, including DBP, has been suspected to have carcinogenic properties [11], to act as enviromental endocrine disruptors [12,13] and to exhibit developmental toxicity [14].

Nowadays, many government agencies worldwide, including the European Environment Agency, the U.S. EPA and the Chinese Ministry of Health have set a maximum residue limit (MRL) for DBP residues (300 ng/mL). Most measures to detect DBP are based on chromatography [15–17] or chromatography-mass spectrum techniques [18–20]. The first immunoassay for phthalate ester came from Ius's research in 1993 [21]. He developed a time-resolved fluoroimmunoassay to detect dimethyl phthalate in water. In recent years, Zhang, MC has published immunoassays for various phthalates including diethyl phthalate (DEP) [22], dimethyl phthalate (DMP) [23,24], dipropyl phthalate (DPP) [25], dibutyl phthalate (DBP) [26], and dicyclohexyl phthalate (DCHP) [27–29]. However, most publications above focused on fluoroimmunoassay or water samples. Until now, few immunoassay [30] were found based on monoclonal antibodies.

In this study, a specific monoclonal antibody against DBP was prepared and an enzyme-linked immunosorbent assay (ELISA) was established to detect the presence of DBP in liquor. Real liquor samples were analyzed with the proposed ELISA, which showed good correspondence with liquid chromatography-mass spectrum results.

## 2. Materials and Methods

### 2.1. Reagents

Phthalate ester standards were obtained from Sinopharm Chemical Reagent Company (Beijing, China). Bovine serum albumin (BSA, MW 67,000) and ovalbumin (OVA, MW 45,000) were purchased from Boao Biotechnology Company (Shanghai, China). HRP-labeled goat anti-mouse immunoglobulin and 3,3′,5,5′-tetramethylbenzidine (TMB) were obtained from Sigma (St. Louis, MO, USA). HPLC grade ethyl alcohol was purchased from J. T. Baker Company (Deventer, The Netherlands). All the other reagents were of analytical grades or better. Purified water from Millipore (resistivity 18.2 MΩ/cm) was used throughout the experiments.

### 2.2. Buffer Solutions

Phosphate-buffered saline (PBS, pH 7.4) was 10 mM sodium phosphate solution. Detergent was prepared by adding 0.05% (v/v) tween 20 in PBS. Blocking solution contained 0.1% gelatin (m/v) in PBS. Carbonate buffer (50 mM, pH 9.6) was used as coating buffer. Substrate solution was prepared by resolving TMB in glycol (0.06%, v/v) and the prepared TMB solution was mixed with citrate-acetate buffer (pH 5.5) at a ratio of 1:5 (v/v) in field use (ready-to-mix TMB substrate). All the solutions above were stored at 4 °C for use.

### 2.3. Monoclonal Antibody Production

4-Nitrophthalic acid (4-NPA) was used to synthesize di-*n*-butyl-4-aminophthalate (DBaP) as described in our previous report [31]. Complete antigens of BSA-DBaP and OVA-DBaP were respectively prepared. BSA conjugates were used to immunize mice and OVA conjugates were used as coating antigen. A positive mouse was sacrificed for cell fusion after five immunizations. Following three cycles of hybridoma selection, a cell line was chosen and expanded to produce a monoclonal antibody. The whole process related to antibody preparation was based on that described in a previous publication [32].

### 2.4. Establishment of ELISA for DBP Analysis

Checkerboard assays were conducted to optimize the concentrations of antibody and coating antigen [32]. Assay buffer, which is used to dilute the sample, plays an important role in ELISA analysis. To evaluate the effect of the alcohol content of wine on ELISA performance, ethanol was chosen as the co-solvent. Various fractions of ethanol content in PBS (10%, 20%, 30% and 40% by volume fraction) were tested. Additionally, ionic strength of assay buffer was optimized by varying the content of sodium chloride in PBS (1%, 3%, 6% and 10% by mass fraction). Optical density (OD) value of each well was read by a micro-titer plate detector (Multiskanmicroplate reader, Thermo Labsystem, Helsinki, Finland).

Under the optimized conditions, series of DBP standards were analyzed. An inhibition curve was plotted based on OD values and DBP concentrations. The concentration of DBP resulting 50% inhibition of the maximum OD value was set as IC_50_ value, which was obtained based on the inhibition curve. Similarly, the IC_50_ values of DBP analogues were determined. Cross-reactivity (CR) for each DBP analogue was calculated with an equation described previously [31]:

(1) $$\text{CR}\%=(\text{I}{\text{C}}_{50}\;\text{value of DBP})/(\text{I}{\text{C}}_{50}\;\text{value of related compound}).$$

### 2.5. Recovery Tests

Considering the widespread presence of phthalate esters, an alcoholic simulant was prepared for recovery tests. The simulant was prepared using water and ethyl alcohol at a volumetric mixture ratio of 1:1(v/v). Fortified samples of the simulant at two concentrations (100 and 300 ng/mL) were used to evaluate this ELISA system.

### 2.6. Sample Analysis

Several liquor samples, purchased from a local market, were detected using the ELISA we developed. In addition, the samples were also analyzed with a linear ion trap-orbitrap hybrid mass spectrometer (MS) at high resolution (LTQ-Oribitrap XL, Thermo Scientific, Waltham, MA, USA). The conditions for DBP analysis with the LTQ-Oribitrap-MS were as follows: the parent ion of DBP was *m*/*z* 301.1401; two resulting daughter ions were *m*/*z* 245.0719 and *m*/*z* 171.09. All data were calculated using the Origin software 8.0 (OriginLab, Northampton, MA, USA).

## 3. Results and Discussion

### 3.1. Development of ELISA

**Figure 1.**
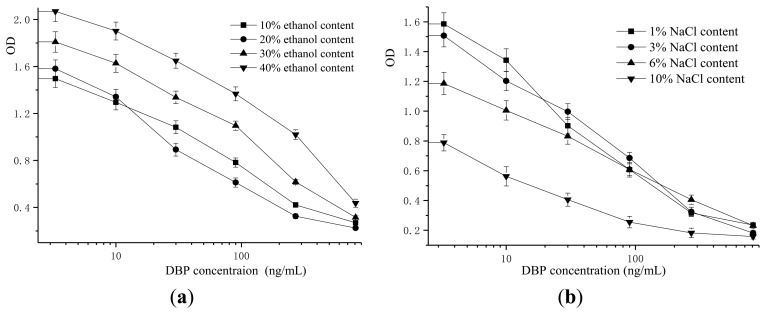
Optimization of assay buffer for ELISA system. (**a**) Effects from ethanol content on performance of ELISA; (**b**)-Effects from NaCl content on performance of ELISA. Each point of inhibition curve represents five replicates in analysis.

Based on the results of checkerboard determination, the concentration of the antigen OVA-DBaP used for coating was 0.1 μg/mL, while the optimum concentration of the antibody was 0.03 μg/mL. Ethanol, was used as co-solvent in the assay buffer to enhance the solubility of DBP. As shown in Figure 1(a), the ethanol content had a large effect on the maximum OD (the value for control wells). Increasing the fraction of ethanol led to an obvious increase in the maximum OD. The lowest IC_50_ value was obtained when the ethanol content was 20% (v/v) in assay buffer.

Ionic strength in the assay buffer greatly affects the binding between antigen and antibody. An appropriate ionic strength in the reaction system is very useful in limiting non-specific antibody binding. From Figure 1(b), a decrease of the maximum OD value was found with the increasing ionic strength of the assay buffer. The maximum OD value was less than 1.0 when the percentage composition of sodium chloride in assay buffer was up to 10% (m/v). Lowest IC_50_ value with a suitable maximum OD value was obtained when the sodium chloride content was 3% (m/v) in assay buffer. Thus, PBS buffer containing 20% (v/v) ethanol and 3% (m/v) sodium chloride was used as assay buffer in our following experiments.

**Figure 2.**
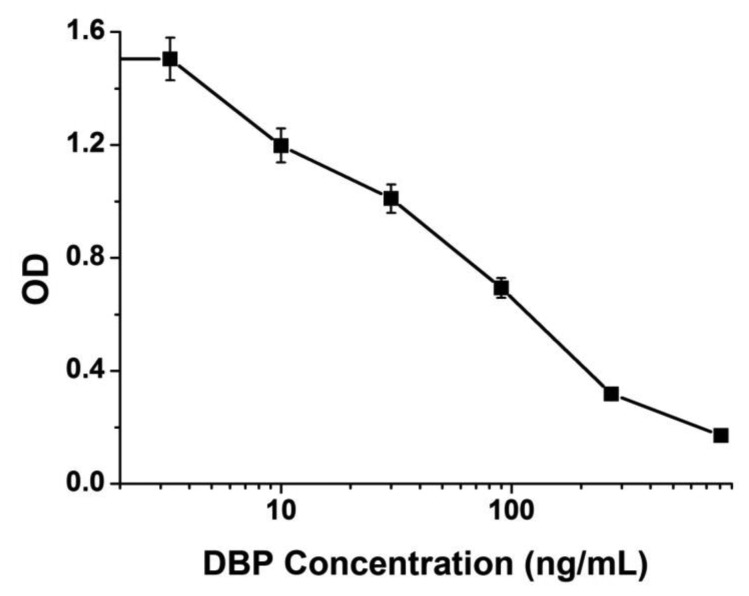
Standard inhibition curve for ELISA analysis of DBP. Each point of inhibition curve represents eight replicates in analysis. The IC_50_ value was 33.6 ± 2.5 ng/mL and the linear range was 5–250 ng/mL.

An “s” shaped curve was created under the optimized conditions above (Figure 2). The IC_50_ value was calculated as 33.6 ± 2.5 ng/mL based on detection of DBP standards. The linear range was 5–250 ng/mL. The sensitivity was set to produce 20% inhibition of the maximum OD value and it was calculated as 8.6 ng/mL for this indirect competitive ELISA.

### 3.2. Cross-Reaction Tests

Eleven phthalate esters and haptens were tested for cross-reaction using the optimized ELISA. As shown in Table 1, this immunoassay system has highly specificity to DBP with little cross-reactivity to other phthalate esters. The related compounds including DIBP (di-isobutyl phthalate), benzyl butyl phthalate (BBP), di(2-ethylhexyl)phthalate (DEHP) and hapten (DBaP) were poorly recognized by this ELISA with low cross-reactivity 4.8%, 2.6%, 2.2%, 2.5% respectively. Little cross-reactivity (<1%) was found for the seven other tested compounds.

The chemical structure of members of the phthalate ester family is very similar. For example, the fine distinctions between DOP (di-*n*-octyl phthalate) and DBP lie in the length of the ester linkage. However, the prepared monoclonal antibody here distinguishes DBP very well. Similarly, a previously reported antibody against diethyl phthalate (DEP) also showed little cross-reactivity to other members of phthalate ester family (<8%) [22]. One possible explanation is that the antibody can differentiate the length of ester linkage in the benzene ring.

### 3.3. Evaluation of DBP Detection in Liquor

The samples of fortified liquor simulant were diluted 2.5-fold with PBS and then analyzed using the proposed ELISA. The detection results were summarized in Table 2. The recovery rate of lower-level fortified samples (100 ng/mL) was 87.7% ± 6.9% with a coefficient of variation (CV) of 8.4% (inter-day tests) and 12.8% (intra-day tests). For samples at higher fortification level (300 ng/mL), the recovery rate was 94.5% ± 5.7% with CV values of 7.1% (inter-day tests) and 9.7% (intra-day tests).

In view of the dilution factors and sensitivity value (8.6 ng/mL), the detection of limit (LOD) of this ELISA for liquor samples was 21.5 ng/mL. Three liquor samples (S1, S2 and S7) were found DBP residues using this ELISA while all the liquor samples were confirmed to contain DBP residues ranging from 3.12 ng/mL to 118.2 ng/mL using LTQ-Orbitrap MS (Table 3). This immunoassay successfully told DBP presence in liquor samples with residue level higher than 21.5 ng/mL. Considering the MRL requirements (300 ng/mL) in food, our proposed ELISA could be applied to screen DBP residues in liquor.

## 4. Conclusions

A specific monoclonal antibody against DBP was prepared and used to develop an ELISA. With simple dilution, liquor samples could be successfully analyzed using this proposed ELISA. The detection results showed good correlation with those from liquid chromatography tandem mass spectrometry. This ELISA exhibits satisfactory performance in DBP analysis with LOD value of 21.5 ng/mL and shows great potential for liquor screening.

## Figures and Tables

**Table 1.** Cross reactivity results under the optimized ELISA conditions.

**Compound**	**Abbreviation**	**IC_50_ ng/mL**	**CR%**
di-*n*-butyl phthalate	DBP	33.6 ± 2.5	100
di-*n*-octyl phthalate	DOP	>5,000	<1
di-*n*-hexyl phthalate	DNHP	>5,000	<1
dimethyl phthalate	DMP	>5,000	<1
bis(2-*n*-butoxyethyl)phthalate	DBEP	>5,000	<1
bis (2-ethoxyethyl) phthalate	DEEP	>5,000	<1
dipentyl phthalate	DPP	>5,000	<1
benzyl butyl phthalate	BBP	1,315 ± 25.4	2.6
dicyclohexyl phthalate	DCHP	>5,000	<1
di-isobutyl phthalate	DIBP	697 ± 12.5	4.8
di(2-ethylhexyl)phthalate	DEHP	1,525 ± 18.2	2.2
4-nitrophthalic acid	4-NPA	>5,000	<1
di-*n*-butyl 4-aminophthalate	DBaP	1,348 ± 21.4	2.5

**Table 2.** Recovery results for fortified liquor simulant.

**Samples**	**Spiked Level (ng/mL)**	**Average** **Recovery (%)**	**CV (%)**		

			**Inter-Day (n** = **6)**	**Intra-Day (n** = **3)**
Liquor	100	87.7 ± 6.9	8.4	12.8
Simulants	300	94.5 ± 5.7	7.1	9.7

**Table 3.** Detection of DBP residues in marketed liquor samples.

**Samples**	**Alcoholic Strength Vol. (%)**	**DBP Residue Level (ng/mL)**	

		ELISA*	**LTQ-Orbitrap MS**
S1	52	27.2	24.31
S2	60	123.4	118.2
S3	53	ND.	8.65
S4	51	ND.	5.21
S5	54	ND.	12.01
S6	52	ND.	3.12
S7	52	72.7	65.8
S8	46	ND.	4.53
S9	38	ND.	13.54

* ND means not detectable.

## References

[1] Wang W., Zhang Y., Wang S., Fan C.Q., Xu H. (2012). Distributions of phthalic esters carried by total suspended particulates in Nanjing, China. Environ. Monit. Assess..

[2] Sendon R., Sanches-Silva A., Bustos J., Martin P., Martinez N., Cirugeda M.E. (2012). Detection of migration of phthalates from agglomerated cork stoppers using HPLC-MS/MS. J. Sep. Sci..

[3] Li X., Xiong W., Lin H., Zhuo L., Lv S., Tang X., Chen M., Zou Z., Lin Z., Qiu B., Chen G. (2013). Analysis of 16 phthalic acid esters in food simulants from plastic food contact materials by LC-ESI-MS/MS. J. Sep. Sci..

[4] Jen J.F., Liu T.C. (2006). Determination of phthalate esters from food-contacted materials by on-line microdialysis and liquid chromatography. J. Chromatogr. A.

[5] Cinelli G., Avino P., Notardonato I., Centola A., Russo M.V. (2013). Rapid analysis of six phthalate esters in wine by ultrasound-vortex-assisted dispersive liquid–liquid micro-extraction coupled with gas chromatography-flame ionization detector or gas chromatography–ion trap mass spectrometry. Anal. Chim. Acta.

[6] Russo M.V., Notardonato I., Cinelli G., Avino P. (2012). Evaluation of an analytical method for determining phthalate esters in wine samples by solid-phase extraction and gas chromatography coupled with ion-trap mass spectrometer detector. Anal. Bioanal. Chem..

[7] Guo Z., Wei D., Wang M., Wang S. (2010). Determination of six phthalic acid esters in orange juice packaged by PVC bottle using SPE and HPLC-UV: Application to the migration study. J. Chromatogr. Sci..

[8] Wu P.G., Yang D.J., Shen X.H., Pan X.D., Wang L.Y., Zhang J., Tan Y., Feng L., Ying Y. (2012). The determination of phthalic acid esters in edible vegetable oils by gas chromatography-mass spectrometry. Zhejiang Yu Fang Yi Xue.

[9] Casajuana N., Lacorte S. (2004). New methodology for the determination of phthalate esters, bisphenol A, bisphenol A diglycidyl ether, and nonylphenol in commercial whole milk samples. J. Agric. Food Chem..

[10] Xu G., Li F., Wang Q. (2008). Occurrence and degradation characteristics of dibutyl phthalate (DBP) and di-(2-ethylhexyl) phthalate (DEHP) in typical agricultural soils of China. Sci. Total Environ..

[11] Carneiro P.A., Umbuzeiro G.A., Oliveira D.P., Zanoni M.V. (2010). Assessment of water contamination caused by a mutagenic textile effluent/dyehouse effluent bearing disperse dyes. J. Hazard. Mater..

[12] Kuch B., Kern F., Metzger J.W., von der Trenck K.T. (2010). Effect-related monitoring: Estrogen-like substances in groundwater. Environ. Sci. Pollut. Res. Int..

[13] Asakura H., Matsuto T., Tanaka N. (2007). Analytical study of endocrine-disrupting chemicals in leachate treatment process of municipal solid waste (MSW) landfill sites. Environ. Sci..

[14] Dobrzyńska M.M., Tyrkiel E.J., Pachocki K.A. (2011). Developmental toxicity in mice following paternal exposure to di-N-butyl-phthalate (DBP). Biomed. Environ. Sci..

[15] Adeniyi A.A., Okedeyi O.O., Yusuf K.A. (2011). Flame ionization gas chromatographic determination of phthalate esters in water, surface sediments and fish species in the Ogun river catchments, Ketu, Lagos, Nigeria. Environ. Monit. Assess..

[16] Niu Z., Ye X., Fang L., Xue Q., Sun Z. (2006). Determination of phthalic acid esters in textiles by solid phase extraction-gas chromatography. Chin. J. Chrom..

[17] Niu H., Cai Y., Shi Y., Wei F., Mou S., Jiang G. (2007). Cetyltrimethylammonium bromide-coated titanate nanotubes for solid-phase extraction of phthalate esters from natural waters prior to high-performance liquid chromatography analysis. J. Chromatogr. A.

[18] Blair J.D., Ikonomou M.G., Kelly B.C., Surridge B., Gobas F.A. (2009). Ultra-trace determination of phthalate ester metabolites in seawater, sediments, and biota from an urbanized marine inlet by LC/ESI-MS/MS. Environ. Sci. Technol..

[19] Cacho J.I., Campillo N., Vinas P., Hernandez-Cordoba M. (2012). Determination of alkylphenols and phthalate esters in vegetables and migration studies from their packages by means of stir bar sorptive extraction coupled to gas chromatography-mass spectrometry. J. Chromatogr. A.

[20] Penalver A., Pocurull E., Borrull F., Marce R.M. (2000). Determination of phthalate esters in water samples by solid-phase microextraction and gas chromatography with mass spectrometric detection. J. Chromatogr. A.

[21] Ius A., Bacigalupo M.A., Meroni G., Pistillo A., Roda A. (1993). Development of a time-resolved fluoroimmunoassay for phthalate esters in water. Fresenius J. Anal. Chem..

[22] Zhang M., Cong Y., Sheng Y., Liu B. (2010). A direct competitive enzyme-linked immunosorbent assay by antibody coated for diethyl phthalate analysis. Anal. Biochem..

[23] Zhang M., Liu S., Zhuang H., Hu Y. (2012). Determination of dimethyl phthalate in environment water samples by a highly sensitive indirect competitive ELISA. Appl. Biochem. Biotechnol..

[24] Zhang M., Liu B., Cong Y., Liu S., Hu Y. (2011). Development of highly specific fluorescence immunoassay and enzyme-linked immunosorbent assay for detection of dimethyl phthalate in water samples. Food Agric. Immunol..

[25] Zhang M., Hu Y., Liu S., Cong Y., Liu B., Wang L. (2012). A highly sensitive enzyme-linked immunosorbent assay for the detection of dipropyl phthalate in plastic food contact materials. Food Agric. Immunol..

[26] Zhang M.C., Wang Q.E., Zhuang H.S. (2006). A novel competitive fluorescence immunoassay for the determination of dibutyl phthalate. Anal. Bioanal. Chem..

[27] Zhang M., Sheng Y. (2010). An indirect competitive fluorescence immunoassay for determination of dicyclohexyl phthalate in water samples. J. Fluoresc..

[28] Zhang M.C., Zhuang H.S., Lang Q. (2006). Study of dicyclohexyl phthalate on preparation and characterization of artificial antigen. Wei Sheng Yan Jiu/J. Hyg. Res..

[29] Zhang M., Wang Y., Yu X., Hu Y., Liu S. (2013). Rapid monitoring of dicyclohexyl phthalate in foods using the direct competitive ELISA. Food Agric. Immunol..

[30] Li L., Zhou Y., Li Y.-S., Feng X.-L., Song J., Liu Y.-Y., Gao S.-Q., Zhang Y.-Y., Li Z.-H., Wang G.-M. (2012). Preparation of an antigen and development of a monoclonal antibody against mono-butyl phthalate (MBP). Food Agric. Immunol..

[31] Kuang H., Xu L.G., Cui G., Ma W., Xu C.L. (2010). Development of determination of di-n-octyl phthalate (DOP) residue by an indirect enzyme-linked immunosorbent assay. Food Agric. Immunol..

[32] Deng X.F., Liu L.Q., Ma W.W., Xu C.L., Wang L.B., Kuang H. (2012). Development and validation of a sandwich ELISA for quantification of peanut agglutinin (PNA) in foods. Food Agric. Immunol..

